# Synthesis and Antibacterial Activity of Quaternary Ammonium 4-Deoxypyridoxine Derivatives

**DOI:** 10.1155/2016/3864193

**Published:** 2016-10-05

**Authors:** Nikita V. Shtyrlin, Sergey V. Sapozhnikov, Albina S. Galiullina, Airat R. Kayumov, Oksana V. Bondar, Elena P. Mirchink, Elena B. Isakova, Alexander A. Firsov, Konstantin V. Balakin, Yurii G. Shtyrlin

**Affiliations:** ^1^Kazan (Volga Region) Federal University, Kremlyovskaya St. 18, Kazan 420008, Russia; ^2^Gause Institute of New Antibiotics, Russian Academy of Medical Sciences, B. Pirogovskaya St. 11, Moscow 119021, Russia; ^3^Institute of Physiologically Active Compounds of Russian Academy of Sciences, Severnyi Pr. 1, Chernogolovka, Moscow 142432, Russia

## Abstract

A series of novel quaternary ammonium 4-deoxypyridoxine derivatives was synthesized. Two compounds demonstrated excellent activity against a panel of Gram-positive methicillin-resistant* S. aureus* strains with MICs in the range of 0.5–2 *μ*g/mL, exceeding the activity of miramistin. At the same time, both compounds were inactive against the Gram-negative* E. coli *and* P. aeruginosa *strains. Cytotoxicity studies on human skin fibroblasts and embryonic kidney cells demonstrated that the active compounds possessed similar toxicity with benzalkonium chloride but were slightly more toxic than miramistin. SOS-chromotest in* S. typhimurium* showed the lack of DNA-damage activity of both compounds; meanwhile, one compound showed some mutagenic potential in the Ames test. The obtained results make the described chemotype a promising starting point for the development of new antibacterial therapies.

## 1. Introduction

Growing antibiotic resistance has become a major clinical problem in recent decades and has encouraged many researchers to search for novel antibacterial drugs. In particular, methicillin-resistant* Staphylococcus aureus* (MRSA), vancomycin-resistant enterococci (VRE), and multidrug-resistant* Pseudomonas aeruginosa* are associated with increased rates of illness and death [1].

Since the 1930s, quaternary ammonium compounds (QACs) are widely used for the control of bacterial growth in clinical and industrial environments. Broad-spectrum antimicrobial and antifungal activity [2–5] and surfactant properties have made QACs such as benzalkonium chloride [6], fluomizin [7], miramistin, and cetylpyridinium chloride [8] the favored hygienic adjuncts in disinfectant cleansing formulations, and they have also been increasingly deployed in the treatment of bacterial infections.

According to literature data [7], QACs generally act by disrupting the cytoplasmic and outer membrane lipid bilayers through association of the positively charged quaternary nitrogen with the anionic head groups of acidic phospholipids and interaction of the lipophilic tail with the hydrophobic membrane core. As a result, QACs form mixed-micelle aggregates with hydrophobic membrane components that solubilize membrane and lyse the cells. Bacterial cell lethality occurs through generalized and progressive leakage of cytoplasmic materials [7]. At the same time, other biomolecular complexes within the bacterial cells are potential targets for action of cationic surfactants which can disrupt critical intermolecular interactions and tertiary structures in such highly specific biochemical systems.

In our group, we have systematically studied chemistry and biology of the biologically active pyridoxine derivatives [9–12]. Recently, we have described a wide series of mono- and bis-ammonium derivatives of pyridoxine and 6-hydroxymethyl pyridoxine [13]. Some of the described compounds possess potent antibacterial activity against several Gram-positive pathogens with minimum inhibitory concentrations (MICs) in the range of 4–64 *μ*g/mL. These promising results encouraged us to synthesize and study their 4-deoxypyridoxine analogs, bearing long alkyl substituents at the quaternary nitrogen. The obtained compounds were tested* in vitro* for their ability to inhibit growth of a wide number of Gram-positive and Gram-negative bacterial pathogens. The most active compounds were also studied for their cytotoxicity and genotoxicity. Our special interest was focused on activity of the obtained compounds against the methicillin-resistant* Staphylococcus aureus* pathogens.

## 2. Materials and Methods

### 2.1. Synthetic Procedures

#### 2.1.1. General Information

^1^H and ^13^C NMR spectra were recorded on a Bruker AVANCE 400 spectrometer at operating frequency of 400 and 101.56 MHz, respectively. Chemical shifts were measured with reference to the residual protons of the solvents (DMSO-d_6_, ^1^H, 2.50 ppm, ^13^C, 39.52 ppm; CDCl_3_, ^1^H, 7.26 ppm, ^13^C, 77.16 ppm; СD_3_OD, ^1^H, 4.87 ppm, ^13^C, 49.00 ppm). Coupling constants (*J*) are given in Hertz (Hz). The following abbreviations are used to describe coupling: s = singlet; d = doublet; t = triplet; m = multiplet; q = quartet; br s = broad singlet. Melting points were determined using a Stanford Research Systems MPA-100 OptiMelt melting point apparatus and are uncorrected. For TLC analysis silica gel plates from Sorbfil (Krasnodar, Russia) were used with UV light (254 nm/365 nm) or iron (III) chloride as developing agent. Column chromatography was performed on silica gel (60–200 mesh) from Acros.

HRMS mass spectra were obtained on a quadrupole time-of-flight (qTOF) AB Sciex Triple TOF 5600 mass spectrometer using turbo-ion spray source (nebulizer gas nitrogen, a positive ionization polarity, and needle voltage 5500 V). Recording of the spectra was performed in a TOF MS mode with collision energy of 10 eV, declustering potential of 100 eV, and resolution more than 30 000 full-width half-maximum. Samples with the analytes concentration of 5 *μ*mol/L were prepared by dissolving of the test compounds in a mixture of methanol (HPLC-UV Grade, LabScan) and water (LC-MS Grade, Panreac) with a ratio of 1 : 1.

#### 2.1.2. General Procedure for Preparation of the Quaternary Ammonium Salts** 4a**–**c**

Tertiary amine (1 equiv.) was added to a solution of compound** 3** (1 equiv.) in 30 mL of DMF. The reaction mixture was heated at 50°C for 20 h and then the solvent was evaporated under reduced pressure. The product was recrystallized from acetone : diethyl ether mixture (10 : 1).

#### 2.1.3. N-((5-Acetoxy-4,6-dimethylpyridin-3-yl)methyl)-N,N-dimethyloctan-1-aminium Chloride (**4a**)

The compound was obtained from** 3** (150 mg, 0.70 mmol) and N,N-dimethyloctylamine (0.144 mL, 0.70 mmol) following the general procedure. Yield 54% (140 mg); white solid; mp 169-170°C (dec). ^1^H NMR (400 MHz, CDCl_3_, *δ*, ppm): 0.82 (t, ^3^ *J*_*нн*_ = 6.8 Hz,  $\underline{{CH}_{3}}$C_7_H_14_, 3H); 1.18–1.26 (m, 5СН_2_, 10Н); 1.72 (br s, СН_2_, 2Н); 2.34 (s, СН_3_, 3Н); 2.36 (s, СН_3_, 3Н); 2.43 (s, СН_3_, 3Н); 3.27 (s, 2CH_3_N^+^, 6H); 3.57–3.61 (m, CH_2_N^+^, 2H); 5.29 (s, CH_2_N^+^, 2H); 8.51 (s, *СН*_pyr_, 1Н). ^13^С NMR (100 MHz, CDCl_3_, *δ*, ppm): 14.08 (s, CH_3_); 14.43 (s, CH_3_); 19.68 (s, CH_3_); 20.50 (s, CH_2_); 22.59 (s, CH_2_); 23.00 (s, CH_2_); 26.39 (s, CH_2_); 29.05 (s, CH_2_); 29.26 (s, CH_2_); 31.64 (s, CH_2_); 49.26 (s, CH_3_N^+^); 62.97 (s, CH_2_N^+^); 63.72 (s, CH_2_N^+^); 122.04 (s, *С*_pyr_); 143.03 (s, *С*_pyr_); 145.55 (s, *С*_pyr_); 151.19 (s, *С*_pyr_); 153.83 (s, *С*_pyr_); 168.20 (s, С=O). HRМS-ESI: found [М–Cl]^+^ 335.2693, C_20_H_35_N_2_O_2_. Calculated [М–Cl]^+^ 335.2693.

#### 2.1.4. N-((5-Acetoxy-4,6-dimethylpyridin-3-yl)methyl)-N,N-dimethyldodecan-1-aminium Chloride (**4b**)

The compound was obtained from** 3** (175 mg, 0.82 mmol) and N,N-dimethyldodecylamine (0.222 mL, 0.82 mmol) following the general procedure. Yield 50% (175 mg); white solid; mp 169-170°C (dec). ^1^H NMR (400 MHz, CDCl_3_, *δ*, ppm): 0.84 (t, ^3^ *J*_*нн*_ = 6.8 Hz,  $\;\underline{{CH}_{3}}$C_11_H_22_, 3H); 1.20–1.28 (m, 9СН_2_, 18Н); 1.73 (br s, СН_2_, 2Н); 2.35 (s, СН_3_, 3Н); 2.37 (s, СН_3_, 3Н); 2.44 (s, СН_3_, 3Н); 3.28 (s, 2CH_3_N^+^, 6H); 3.57–3.61 (m, CH_2_N^+^, 2H); 5.29 (s, CH_2_N^+^, 2H); 8.51 (s, *СН*_pyr_, 1Н). ^13^С NMR (100 MHz, CDCl_3_, *δ*, ppm): 14.19 (s, CH_3_); 14.46 (s, CH_3_); 19.72 (s, CH_3_); 20.52 (s, CH_2_); 22.74 (s, CH_2_); 23.04 (s, CH_2_); 26.42 (s, CH_2_); 29.35 (s, CH_2_); 29.38 (s, CH_2_); 29.43 (s, CH_2_); 29.50 (s, CH_2_); 29.64 (s, CH_2_); 49.31 (s, CH_3_N^+^); 63.03 (s, CH_2_N^+^); 63.74 (s, CH_2_N^+^); 122.01 (s, C_pyr_); 143.06 (s, C_pyr_); 145.59 (s, C_pyr_); 151.20 (s, C_pyr_); 153.91 (s, C_pyr_); 168.23 (s, С=O). HRМS-ESI: found [М–Cl]^+^ 391.3319, C_25_H_43_N_2_O_2_. Calculated [М–Cl]^+^ 391.3319.

#### 2.1.5. N-((5-Acetoxy-4,6-dimethylpyridin-3-yl)methyl)-N,N-dimethyloctadecan-1-aminium Chloride (**4c**)

The compound was obtained from** 3** (150 mg, 0.70 mmol) and N,N-dimethyloctadecylamine (0.261 mL, 0.70 mmol) following the general procedure. Yield 53% (190 mg); white solid; mp 159-160°C (dec). ^1^H NMR (400 MHz, CDCl_3_, *δ*, ppm): 0.85 (t, ^3^ *J*_*нн*_ = 6.7 Hz,  $\;\underline{{CH}_{3}}$C_17_H_34_, 3H); 1.21–1.29 (m, 15СН_2_, 30Н); 1.74 (br s, СН_2_, 2Н); 2.36 (s, СН_3_, 3Н); 2.39 (s, СН_3_, 3Н); 2.46 (s, СН_3_, 3Н); 3.29 (s, 2CH_3_N^+^, 6H); 3.57–3.61 (m, CH_2_N^+^, 2H); 5.30 (s, CH_2_N^+^, 2H); 8.50 (s, *СН*_pyr_, 1Н). ^13^С NMR (100 MHz, CDCl_3_, *δ*, ppm): 14.22 (s, CH_3_); 14.51 (s, CH_3_); 19.75 (s, CH_3_); 20.55 (s, CH_2_); 22.79 (s, CH_2_); 23.07 (s, CH_2_); 26.44 (s, CH_2_); 29.39 (s, CH_2_); 29.46 (s, CH_2_); 29.55 (s, CH_2_); 29.69 (s, CH_2_); 29.76 (s, CH_2_); 29.80 (s, CH_2_); 32.02 (s, CH_2_); 49.36 (s, CH_3_N^+^); 63.10 (s, CH_2_N^+^); 63.79 (s, CH_2_N^+^); 122.01 (s, C_pyr_); 143.13 (s, C_pyr_); 145.64 (s, C_pyr_); 153.18 (s, C_pyr_); 153.98 (s, C_pyr_); 168.26 (s, С=O). HRМS-ESI: found [М–Cl]^+^ 475.4258, C_30_H_55_N_2_O_2_. Calculated [М–Cl]^+^ 475.4258.

#### 2.1.6. General Procedure for Preparation of** 5a**–**c**

A mixture of quaternary ammonium salt** 4a**–**c** (1 equiv.) and 1 mL of concentrated HCl in 20 mL of water was stirred at 60°C for 24 h. The solvent was evaporated under reduced pressure to obtain** 5a**–**c** in quantitative yield.

#### 2.1.7. 5-((Octyldimethylammonio)methyl)-3-hydroxy-2,4-dimethylpyridin-1-ium Dichloride (**5a**)

The compound was obtained from** 4a** (100 mg, 0.27 mmol) following the general procedure. Yield quantitative (0.99 mg); white solid; mp 186–188°C (dec.). ^1^H NMR (400 MHz, DMSO-d_6_, *δ*, ppm): 0.87 (t, ^3^ *J*_*нн*_ = 6.8 Hz,  $\;\underline{{CH}_{3}}$C_11_H_22_, 3H); 1.27–1.31 (m, 5СН_2_, 5Н); 1.77 (br s, СН_2_, 2Н); 2.50 (s, СН_3_, 3Н); 2.68 (s, СН_3_, 3Н); 2.99 (s, 2CH_3_N^+^, 6H); 3.42–3.46 (m, CH_2_N^+^, 2H); 4.78 (s, CH_2_N^+^, 2H); 8.49 (s, *СН*_pyr_, 1Н); 10.96 (s, OH, 1H). ^13^С NMR (100 MHz, DMSO-d_6_, *δ*, ppm): 13.94 (s, CH_3_); 14.87 (s, CH_3_); 15.43 (s, CH_3_); 21.83 (s, CH_2_); 22.03 (s, CH_2_); 25.84 (s, CH_2_); 28.41 (s, CH_2_); 28.48 (s, CH_2_); 31.14 (s, CH_2_); 48.66 (s, CH_3_N^+^); 60.43 (s, CH_2_N^+^); 64.48 (s, CH_2_N^+^); 124.81 (s, C_pyr_); 136.27 (s, C_pyr_); 142.15 (s, C_pyr_); 152.87 (s, C_pyr_). HRМS-ESI: found [М–Cl]^+^ 293.2587, C_18_H_34_N_2_O. Calculated [М–Cl]^+^ 293.2587.

#### 2.1.8. 5-((Dodecyldimethylammonio)methyl)-3-hydroxy-2,4-dimethylpyridin-1-ium Dichloride (**5b**)

The compound was obtained from** 4b** (100 mg, 0.23 mmol) following the general procedure. Yield quantitative (0.99 mg); white solid; mp 187-188°C (dec.). ^1^H NMR (400 MHz, CD_3_OD, *δ*, ppm): 0.88 (t, ^3^ *J*_*нн*_ = 6.7 Hz,  $\;\underline{{CH}_{3}}$C_11_H_22_, 3H); 1.28–1.42 (m, 18СН_2_, 9Н); 1.90 (br s, СН_2_, 2Н); 2.60 (s, СН_3_, 3Н); 2.70 (s, СН_3_, 3Н); 3.09 (s, 2CH_3_N^+^, 6H); 3.50 (br s, CH_2_N^+^, 2H); 4.82 (s, CH_2_N^+^, 2H); 8.49 (s, *СН*_pyr_, 1Н). ^13^С NMR (100 MHz, CD_3_OD, *δ*, ppm): 14.43 (s, CH_3_); 15.47 (s, CH_3_); 15.63 (s, CH_3_); 23.73 (s, CH_2_); 23.81 (s, CH_2_); 27.50 (s, CH_2_); 30.32 (s, CH_2_); 30.46 (s, CH_2_); 30.58 (s, CH_2_); 30.65 (s, CH_2_); 30.74 (s, CH_2_); 33.06 (s, CH_2_); 50.29 (s, CH_3_N^+^); 62.69 (s, CH_2_N^+^); 67.40 (s, CH_2_N^+^); 126.59 (s, C_pyr_); 136.93 (s, C_pyr_); 143.83 (s, C_pyr_); 148.57 (s, C_pyr_); 155.46 (s, C_pyr_). HRМS-ESI: found [М–H–2Cl]^+^ 349.3213, C_22_H_42_N_2_O. Calculated [М–H–2Cl]^+^ 349.3213.

#### 2.1.9. 5-((Dimethyl(octadecyl)ammonio)methyl)-3-hydroxy-2,4-dimethylpyridin-1-ium Dichloride (**5c**)

The compound was obtained from** 4c** (100 mg, 0.20 mmol) following the general procedure. Yield quantitative (0.99 mg); white solid; mp 185–187°C (dec.). ^1^H NMR (400 MHz, DMSO-d_6_, *δ*, ppm): 0.85 (t, ^3^ *J*_*нн*_ = 6.7 Hz,  $\;\underline{{CH}_{3}}$C_17_H_34_, 3H); 1.23–1.33 (m, 15СН_2_, 30Н); 1.77 (br s, СН_2_, 2Н); 2.50 (s, СН_3_, 3Н); 2.68 (s, СН_3_, 3Н); 2.99 (s, 2CH_3_N^+^, 6H); 3.42–3.46 (m, CH_2_N^+^, 2H); 4.77 (s, CH_2_N^+^, 2H); 8.49 (s, CH_pyr_, 1Н); 10.94 (s, OH, 1H). ^13^С NMR (100 MHz, DMSO-d_6_, *δ*, ppm): 13.94 (s, CH_3_); 14.86 (s, CH_3_); 15.46 (s, CH_3_); 21.85 (s, CH_2_); 22.08 (s, CH_2_); 25.85 (s, CH_2_); 28.56 (s, CH_2_); 28.68 (s, CH_2_); 28.79 (s, CH_2_); 29.03 (s, CH_2_); 31.27 (s, CH_2_); 48.66 (s, CH_3_N^+^); 60.42 (s, CH_2_N^+^); 64.50 (s, CH_2_N^+^); 124.81 (s, C_pyr_); 136.29 (s, C_pyr_); 142.17 (s, C_pyr_); 152.87 (s, C_pyr_). HRМS-ESI: found [М–H–2Cl]^+^ 433.4152, C_28_H_54_N_2_O. Calculated [М–H–2Cl]^+^ 433.4152.

### 2.2. Biological Experiments

#### 2.2.1. Antibacterial Activity

The antibacterial activity of compounds was evaluated on the number of Gram-positive (*Staphylococcus aureus* ATCC® 29213*™*,* Staphylococcus epidermidis* (clinical isolate),* Micrococcus luteus* (clinical isolate), and* Bacillus subtilis* 168) and Gram-negative bacteria (*Escherichia coli* АТСС 25922*™*,* Pseudomonas aeruginosa* АТСС 27853*™*, and* Salmonella typhimurium TA100*). Clinical isolates of* Staphylococcus epidermidis* and* Micrococcus luteus* were obtained from the Kazan Institute of Epidemiology and Microbiology (Kazan, Russia). The antibacterial activity of compounds** 4c** and** 5c **was additionally evaluated on* Staphylococcus aureus* ATCC 700699*™* and a number of clinical isolates:* Staphylococcus aureus* 100 MRSA,* Staphylococcus aureus* 5 MRSA,* Staphylococcus aureus* 6 MRSA,* Staphylococcus aureus *3797 MRSA,* Staphylococcus aureus *3798 MRSA,* Staphylococcus aureus* 4603 MRSA,* Staphylococcus haemolyticus* 161,* Staphylococcus haemolyticus* 1025,* Staphylococcus haemolyticus* 602,* Staphylococcus haemolyticus* 585,* Staphylococcus epidermidis* 681,* Staphylococcus epidermidis *9,* Enterococcus faecalis* 560,* Enterococcus faecium *569,* Escherichia coli *396, and* Pseudomonas aeruginosa *43.

The MICs of compounds were determined by the broth microdilution method in Mueller-Hinton (MH) broth (pH = 7.3) in 96-well plates. The double-dilution series of compounds with the final concentrations ranging from 1000 to 0.5 *μ*g/mL in the initial screening experiment and 64–0.5 *μ*g/mL in the evaluation of** 4c** and** 5c** on clinical isolate strains were used. The bacterial suspension (2–9 × 10^4^ CFU/mL) 200 *μ*L aliquots were seeded into 96-well plates and their incubation was followed. The MIC was determined as the lowest concentration of compound for which no visible bacterial growth could be observed after 24 h of incubation at 37°C.

#### 2.2.2. Cytotoxic Activity

Human skin fibroblasts (HSFs) were isolated from the skin explant according to the conventional protocol [17]. HEK 293 (human embryonic kidney) cells were obtained from the ATCC collection. HSFs cells were cultured in the minimum essential medium Eagle (*α*-MEM) supplemented with 10% fetal bovine serum, 2 mM L-glutamine, 100 *μ*g/mL streptomycin, and 100 U/mL penicillin under standard conditions (37°C, 5% CO_2_ atmosphere). HEK 293 cells were grown in the same conditions, but in the Dulbecco's modified Eagle's medium (DMEM). Adhered cells were collected from the culture flask by detaching them with trypsin-EDTA solution. Suspended cells were washed by centrifugation at 200 g in PBS.

Cytotoxic concentrations (IC_50_) of compounds were determined with the use of MTT assay. Cells were preseeded in 96-well plate at the density of 1000–2000 cells per well and cultured with adding a series of diluted water solutions of compounds for 3 days under standard conditions. Culture medium in the plate was then replaced by the fresh one supplemented with 0.5 mg/mL MTT and additionally kept for 4 h to allow for reduction of MTT into colored product (formazan) by metabolically active cells. Optical absorbance of produced formazan, proportional to viable cell number, was registered on Infinite 200 PRO analyzer at 550 nm.

#### 2.2.3. Genotoxicity

*S. typhimurium* strain TA100 [18] was grown overnight in 5 mL of LB medium, diluted 4 times by prewarmed LB and incubation was continued for 2 h. Cells were harvested, washed once by 1x salt base solution (g/L: sodium citrate × 3Н_2_О − 0.5; К_2_НРО_3_  × 3Н_2_О − 14; КН_2_РО_3_ − 6; (NH_4_)_2_SO_4_ − 1; MgSO_4_  × 7Н_2_О − 0.5) and resuspended in 6 mL of 1x salt base. 100 *μ*L of bacterial suspension was mixed with top agar (0.5% agar, 0.5% NaCl, 50 mM L-histidine, 50 mM biotin, pH 7.4, 42°C) in a final volume of 3 mL and the substance to be tested. Each mixture was then seeded onto the minimal agar plates (1.5% agar in the 1x salt base supplemented with 0.5% glucose and ampicillin 10 *μ*g/mL). Next the plates were incubated at 37°C for 72 hours and colonies were counted. Sodium azide (10 *μ*g/mL) was used as a positive control.

The SOS-chromotest was performed by using the* Salmonella typhimurium TA1535/pSK1002* as described in [19]. Briefly, aliquots of 0.5 mL of an overnight culture of the tester strains were diluted in 5 mL of LB medium and then incubated with rigorous agitation in presence of the ficin substances. The Mitomycin C (Sigma) at concentration of 1 *μ*g/mL was used as a positive control. After 4 h of incubation, the cell density (A600) and the *β*-galactosidase activity were measured by Miller's protocol [20] with modifications. Cells were harvested from 0.5 to 1.5 mL of culture liquid and resuspended in 800 *μ*L of Z-buffer [60 mM Na_2_HPO_4_·7H_2_O, 40 mM NaH_2_PO_4_·H_2_O, 10 mM KCl, and 1 mM MgSO_4_·7H_2_O (pH 7.0)] containing additionally 0.005% cetyl trimethylammonium bromide (CTAB) and 50 mM *β*-mercaptoethanol was added. After preincubation at 30°C for 5 min, the reaction was started by adding of 200 *μ*L of 4 mg/mL o-nitrophenyl-*β*-D-galactopyranoside in Z-buffer. When the yellow color appeared, the reaction was stopped by 500 *μ*L of 1 M Na_2_CO_3_. For the blank solution, the Na_2_CO_3_ was added prior to the incubation. The *β*-galactosidase activity was measured at A420 nm. To calculate the Miller units, we used the following formula: [A420/(A600 of 1 : 10 dilution of cells × time of incubation)] × 1000.

## 3. Results and Discussion

**Scheme 1:**
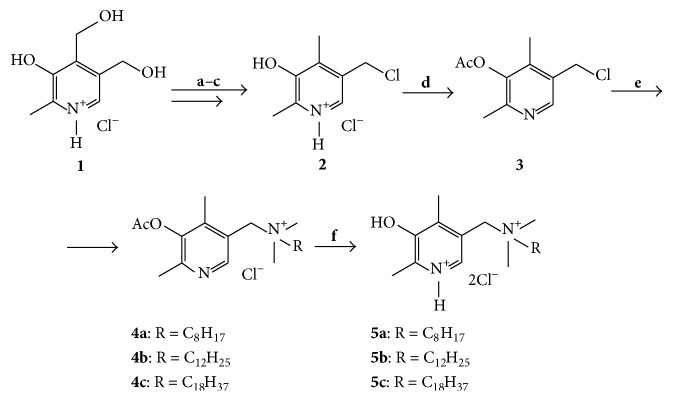
Reagents and conditions: (**a**) Zn, AcOH, reflux [15]; (**b**) HCl, EtOH-H_2_O, 25°C [15]; (**c**) SOCl_2_, DMF, reflux [15]; (**d**) AcCl, NEt_3_, CH_2_Cl_2_, reflux [16]; (**e**) DMF, (CH_3_)_2_RN, 50°C; (**f**) H_2_O, HCl, 25°C.

Key chloride intermediate** 2** was obtained from pyridoxine hydrochloride** 1** according to literature method [15] (Scheme 1). Interaction of** 2** with acetyl chloride in the presence of triethylamine led to acetyl derivative** 3** [16]. The latter was used for alkylation of the tertiary amines in dimethylformamide, which led to the quaternary ammonium salts** 4a**–**c** and, after deacetylation under acidic conditions, to compounds** 5a**–**c**. All attempts to obtain compounds** 5a**–**c** directly from amines and chloride** 2** without protection of hydroxyl group were unsuccessful, probably, due to instability of** 2** under the reaction conditions.

Compounds** 4a**–**c** and** 5a**–**c** were evaluated for antibacterial activity against three Gram-positive and three Gram-negative bacterial strains.  Table 1 shows MICs of the tested compounds in comparison with miramistin and benzalkonium chloride. Two compounds** 4c** and** 5c** with the longest octadecyl substituent demonstrated high antibacterial activity with MICs in the range of 2–32 *μ*g/mL for all pathogens with the exception of* P. aeruginosa*. In general, their activity in this test was comparable with that of the reference drugs.

Compounds** 4c** and** 5c** with promising activities in the primary assay were selected for further evaluation. First, they were studied on an extended panel of clinical Gram-positive and Gram-negative bacterial strains in comparison with miramistin (Table 2). Both compounds exhibited strong activity against all* Staphylococcus *strains and, moreover, compound** 5c** was significantly more active than miramistin. At the same time, compounds** 4c** and** 5c** were inactive against the studied Gram-negative* E. coli *and* P. аeruginosa *strains thus demonstrating a good selectivity for the Gram-positive pathogens. Interestingly, the clinical isolates of* S. aureus* were more sensitive to both** 4c** and** 5c** than* S. aureus *ATCC 29213 cells, while the clinical isolate* E. coli* 396 was not sensitive to** 4c** and** 5c** in contrast to* E. coli* K12. We attribute these effects to the different phenotypic resistance of the strains to antimicrobials [21, 22].

Compounds** 4c** and** 5c** were further examined for their cytotoxicity on human skin fibroblasts (HSF) and human embryonic kidney (HEK 293) cells with the use of MTT assay. According to Table 3, both compounds possessed similar benzalkonium chloride cytotoxicity but were slightly more toxic than miramistin. Despite the satisfactory results obtained in the MTT assay, it should be noted that further studies with the use of lactate dehydrogenase (LDH) release assay or by imaging the uptake of cell-impermeable fluorescent dyes are desirable to more thoroughly assess possible membrane-related toxicity of the described compounds.

Genotoxicity of compounds** 4c** and** 5c** was evaluated by using SOS-chromotest in* Salmonella typhimurium* TA1535/pSK1002 strain as described by Oda et al. [18]. Mitomycin C was used as a positive control. The optical density at 420 nm (OD_420_) was measured, and *β*-galactosidase activity was normalized to the amount of cells estimated from the OD_600_ values. SOS induction factor was calculated as a ratio of *β*-galactosidase activity in the presence of compounds and the solvent control (Table 4). No significant dose-dependent increase more than 2-fold was observed thus indicating the lack of DNA-damage activity of compounds** 4c **and** 5c** under the tested concentrations.

The Ames test was then performed using* Salmonella typhimurium* TA100 [19]. The compounds were tested in concentrations 2, 4, and 8 *μ*g/mL close to their CC_50_ values for* Salmonella *cells. Sodium azide was used as a positive mutagenic control. No increase in the number of revertant colonies as well as no dose dependency was observed for** 4c**, thus suggesting the lack of mutagenicity (Table 5). At the same time,** 5c** caused some dose-dependent increase in the number of revertant colonies. Probably, this suggests some mutagenic potential of this compound.

**Figure 1:**
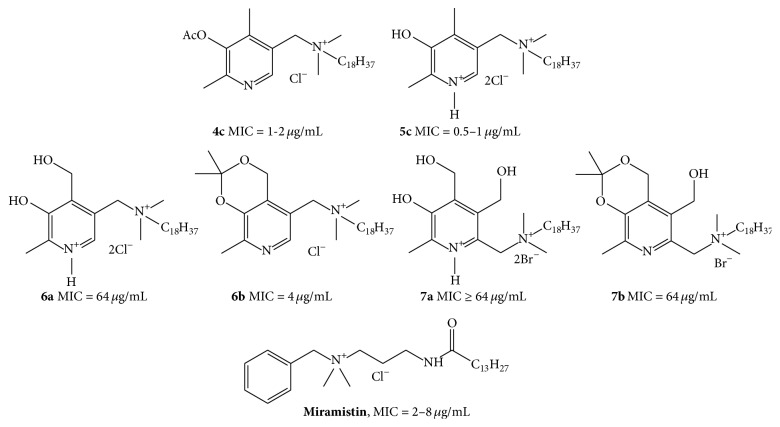
Structures of antibacterial QACs studied in this work (**4c** and** 5c**), their recently reported structural analogs (**6a**,** b** and** 7a**,** b**) [13], and miramistin. MIC values are shown for* S. aureus*.

It was further interesting to compare the obtained results with our recently reported data [13] on antibacterial activity of a series of close structural analogs, the quaternary ammonium salts of pyridoxine and 6-hydroxymethyl pyridoxine (Figure 1).

In that study, we observed a strong correlation between the length of the substituent's chain and antimicrobial activity. The most active compounds had the same octadecyl substituent at the quaternary nitrogen (structures** 6a**,** 6b** and** 7a**,** 7b**) and were bactericidal against several Gram-positive pathogens including* S. aureus*. Compounds** 6a**,** 6b** and** 7a**,** 7b** exhibited a moderate activity against a panel of Gram-positive pathogens (MIC > 4 *μ*g/mL,* S. aureus*), and this activity demonstrated some dependence on compound's lipophilicity. In particular, such a dependency led to activity decrease upon removal of the acetonide protective group.

**Figure 2:**
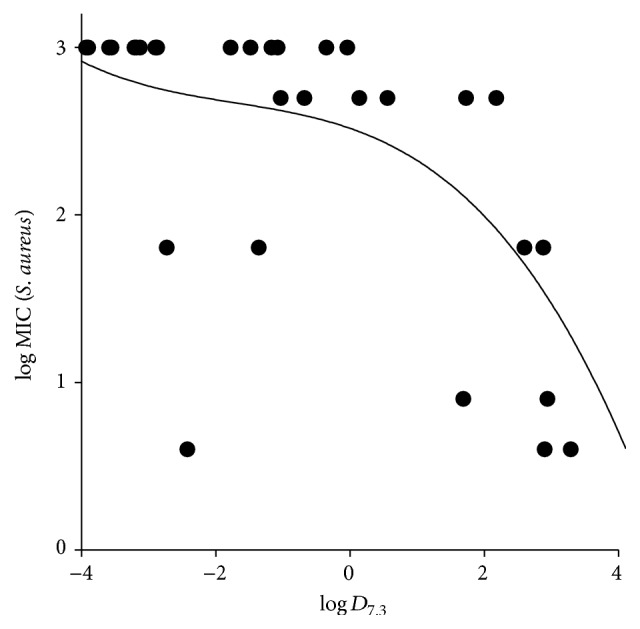
Log MIC (*S. aureus*) versus calculated log⁡*D*_7.3_ [14] relationship for the combined set of the quaternary ammonium pyridoxine derivatives reported in our recent study [13] and in the current paper. A polynomial trend line is shown.

This dependency is more general, though not absolute, for the studied QACs.  Figure 2 demonstrates the relationship between the experimental MICs for* S. aureus* (expressed in a decimal logarithm scale) and calculated log⁡*D* values at pH 7.3 for the combined set of 32 quaternary ammonium pyridoxine derivatives reported in our recent study [13] and in the current paper. Probably, it reflects the important feature of the active compounds essential for their effective interaction with the hydrophobic membrane core of bacterial cells.

In this work, we obtained more active antibacterial agents (MIC in the range of 0.5–2 *μ*g/mL against the studied methicillin-resistant* S. aureus* strains) than those described in the recent paper [13] but with similar selectivity profile and, as may be supposed, similar mechanism of action.

So far, no specific target has been identified for most QACs; it is assumed that the effect is rather generalized than specific to one target. However, as discussed in literature [7], there should be some target specificities, for example, as shown for the bisquaternary bisnaphthalimide MT02 [23], because the activity of QACs toward different bacterial species varies substantially and cannot be explained simply by the structure of cationic charge and hydrophobic portions. It is possible that the most potent agents exert membrane damage, leading to disruption of the cell envelope and arresting intracellular activity by binding targets in the cytoplasm [24].

It can be suggested that all the studied substances interact with bacterial membranes, given their clear structural similarity to other QACs such as miramistin. However, the role of intracellular targets in their antibacterial action remains unclear. Pyridoxine molecule is a well-established cofactor for many enzymes. Therefore, pyridoxine derivatives can participate in many intracellular interactions thus leading to enhanced or more specific antibacterial action. The observed effect of** 5c** in the Ames test may suggest some specific interaction with DNA; however, this hypothesis requires further experimental investigation.

Interestingly, Gram-positive bacteria are generally more sensitive to the obtained compounds than Gram-negative bacteria, which is in agreement with literature data on QACs [7]. This has been attributed to the outer membrane of Gram-negative bacteria, which is absent in Gram-positive strains. Importantly, many antibiotic-resistant staphylococci (such as MRSA or methicillin-resistant* S. epidermidis*) have acquired plasmids such as qacA, qacB, qacC, or qacD, which encode efflux pumps and thus confer resistance determinants to QACs [25]. This suggests that intracellular accumulation of QACs is important for full activity and underscores the importance of the interaction between QACs and intracellular targets for antibacterial activity.

## 4. Conclusion

In this work we have synthesized a small series of novel quaternary ammonium 4-deoxypyridoxine derivatives and studied their antibacterial activity, cytotoxicity, and genotoxicity. Two compounds demonstrate promising* in vitro* antibacterial activity and toxicity when compared with the reference antibacterials miramistin and benzalkonium chloride. The obtained results make the described chemotype a promising starting point for the development of new antibacterial therapies with selective effect on Gram-positive pathogens including their drug-resistant forms.

## Supplementary Material

The Supplementary Information contains synthetic procedures, analytical characteristics of compounds 4a-c and 5a-c, and biological experimental methods associated with this paper.

## Figures and Tables

**Table 1:** * In vitro* antimicrobial activity of the obtained 4-deoxypyridoxine ammonium salts.

Compounds	MICs (*µ*g/mL)					
	Gram-positive bacteria			Gram-negative bacteria		
	*S. aureus*	*S. epidermidis*	*M. luteus*	*E. coli K12*	*S. typhimurium TA100*	*P. aeruginosa*
**4a**	>64	>64	>64	>64	>64	>64
**4b**	>64	32	>64	>64	>64	>64
**4c**	32	2	4	4	8	>64
**5a**	>64	>64	>64	>64	>64	>64
**5b**	>64	>64	>64	>64	>64	>64
**5c**	8	4	8	2	16	>64
Miramistin	4	2	2	32	>64	16
Benzalkonium chloride	2	2	32	4	16	64

**Table 2:** * In vitro* antimicrobial activity of **4c** and **5c** on an extended panel of clinical bacterial pathogens.

Strains	MICs (*µ*g/mL)		
	**4c**	**5c**	Miramistin
*S. aureus 700699 ATCC*	1	1	2
*S. aureus 100 *(MRSA)	2	0.5	2
*S. aureus 5 *(MRSA)	1	0.5	8
*S. aureus 6 *(MRSA)	1	0.5	2
*S. aureus 3797 *(MRSA)	2	1	4
*S. aureus 3798 *(MRSA)	1	0.5	2
*S. aureus 4603 *(MRSA)	2	0.5	4
*S. haemolyticus 161*	2	1	4
*S. haemolyticus 1025*	1	0.5	4
*S. haemolyticus 602*	2	1	4
*S. haemolyticus 585*	4	2	2
*S. epidermidis 681*	4	2	1
*S. epidermidis 9*	4	4	2
*E. faecalis 560*	4	2	2
*E. faecalis 569*	2	4	1
*E. faecalis 101*	4	8	2
*E. coli 396*	>64	>64	32
*P. aeruginosa 43*	>64	>64	64

**Table 3:** Cytotoxicity of the ammonium salts **4c** and **5c**.

Compounds	CC_50_, *µ*g/mL	
	HSF	HEK 293
**4c**	2.88 ± 0.59	2.32 ± 0.75
**5c**	3.25 ± 0.45	2.96 ± 0.9
Miramistin	4.09 ± 0.42	4.08 ± 0.77
Benzalkonium chloride	2.11 ± 0.12	2.04 ± 0.9

**Table 4:** DNA-damage activity of **4c** and **5c** in the SOS-chromotest (ratio, fold increase over the solvent control).

Compounds	Concentration, *μ*g/mL		
	**4**	**20**	**40**
**4c**	0.9 ± 0.32	1.3 ± 0.25	1.4 ± 0.45
**5c**	1.2 ± 0.35	1.5 ± 0.43	1.3 ± 0.53
Mitomycin C	11.7 ± 2.5	—	—

**Table 5:** Mutagenicity of **4c** and **5c** in the Ames test (ratio, fold increase over the solvent control).

Compounds	Concentration, *μ*g/mL		
	2	4	8
**4c**	0.9 ± 0.21	0.6 ± 0.15	0.2 ± 0.12
**5c**	1.8 ± 0.74	2.3 ± 0.53	2.4 ± 0.57
Sodium azide	6.5 ± 2.5	8.3 ± 1.4	9.8 ± 4.6

## References

[1] Nordmann P., Naas T., Fortineau N., Poirel L. (2007). Superbugs in the coming new decade; multidrug resistance and prospects for treatment of *Staphylococcus aureus*, *Enterococcus* spp. and *Pseudomonas aeruginosa* in 2010. *Current Opinion in Microbiology*.

[2] Minbiole K. P. C., Jennings M. C., Ator L. E. (2016). From antimicrobial activity to mechanism of resistance: the multifaceted role of simple quaternary ammonium compounds in bacterial eradication. *Tetrahedron*.

[3] Obłak E., Piecuch A., Krasowska A., Łuczyński J. (2013). Antifungal activity of gemini quaternary ammonium salts. *Microbiological Research*.

[4] Massi L., Guittard F., Levy R., Duccini Y., Géribaldi S. (2003). Preparation and antimicrobial behaviour of gemini fluorosurfactants. *European Journal of Medicinal Chemistry*.

[5] Ohkura K., Sukeno A., Yamamoto K., Nagamune H., Maeda T., Kourai H. (2003). Analysis of structural features of bis-quaternary ammonium antimicrobial agents 4,4′-(*α*,*ω*-polymethylenedithio)bis (1-alkylpyridinium iodide)s using computational simulation. *Bioorganic and Medicinal Chemistry*.

[6] Domagk G. (1935). Eine neue Klasse von Desinfektionsmitteln. *Deutsche Medizinische Wochenschrift*.

[7] Tischer M., Pradel G., Ohlsen K., Holzgrabe U. (2012). Quaternary ammonium salts and their antimicrobial potential: targets or nonspecific interactions?. *ChemMedChem*.

[8] Fromm-Dornieden C., Rembe J.-D., Schäfer N., Böhm J., Stuermer E. K. (2015). Cetylpyridinium chloride and miramistin as antiseptic substances in chronic wound management—prospects and limitations. *Journal of Medical Microbiology*.

[9] Shtyrlin N. V., Lodochnikova O. A., Pugachev M. V. (2010). Theoretical and experimental study on cyclic 6-methyl-2,3,4- tris(hydroxymethyl)pyridin-5-ol acetonides. *Russian Journal of Organic Chemistry*.

[10] Pugachev M. V., Shtyrlin N. V., Sapozhnikov S. V. (2013). Bis-phosphonium salts of pyridoxine: the relationship between structure and antibacterial activity. *Bioorganic and Medicinal Chemistry*.

[11] Shtyrlin N. V., Pavelyev R. S., Pugachev M. V., Sysoeva L. P., Musin R. Z., Shtyrlin Yu. G. (2012). Synthesis of novel 6-substituted sulfur-containing derivatives of pyridoxine. *Tetrahedron Letters*.

[12] Pugachev M. V., Shtyrlin N. V., Sysoeva L. P. (2013). Synthesis and antibacterial activity of novel phosphonium salts on the basis of pyridoxine. *Bioorganic and Medicinal Chemistry*.

[13] Shtyrlin N. V., Sapozhnikov S. V., Koshkin S. A. (2015). Synthesis and antibacterial activity of novel quaternary ammonium pyridoxine derivatives. *Medicinal Chemistry*.

[14] Kah M., Brown C. C. (2008). Log*D*: lipophilicity for ionisable compounds. *Chemosphere*.

[15] Serwa R., Nam T.-G., Valgimigli L. (2010). Preparation and investigation of vitamin B6-derived aminopyridinol antioxidants. *Chemistry—A European Journal*.

[16] Shtyrlin N. V., Vafina R. M., Pugachev M. V. (2016). Synthesis and biological activity of tertiary phosphonium salts based on 3-hydroxypyridine and 4-deoxypyridoxine. *Russian Chemical Bulletin*.

[17] Rittié L., Fisher G. J. (2005). Isolation and culture of skin fibroblasts. *Methods in Molecular Medicine*.

[18] Oda Y., Nakamura S.-I., Oki I., Kato T., Shinagawa H. (1985). Evaluation of the new system (*umu*-test) for the detection of environmental mutagens and carcinogens. *Mutation Research*.

[19] McCann J., Ames B. N. (1976). A simple method for detecting environmental carcinogens as mutagens. *Annals of the New York Academy of Sciences*.

[20] Miller J. H. (1972). *Experiments in Molecular Genetics*.

[21] Cosgrove S. E., Kaye K. S., Eliopoulous G. M., Carmeli Y. (2002). Health and economic outcomes of the emergence of third-generation cephalosporin resistance in *Enterobacter* species. *Archives of Internal Medicine*.

[22] Blair J. M. A., Webber M. A., Baylay A. J., Ogbolu D. O., Piddock L. J. V. (2015). Molecular mechanisms of antibiotic resistance. *Nature Reviews Microbiology*.

[23] González-Bulnes L., Gallego J. (2009). Indirect effects modulating the interaction between DNA and a cytotoxic bisnaphthalimide reveal a two-step binding process. *Journal of the American Chemical Society*.

[24] Locher H. H., Ritz D., Pfaff P. (2010). Dimers of nostocarboline with potent antibacterial activity. *Chemotherapy*.

[25] Paulsen I. T., Brown M. H., Littlejohn T. G., Mitchell B. A., Skurray R. A. (1996). Multidrug resistance proteins QacA and QacB from *Staphylococcus aureus*: membrane topology and identification of residues involved in substrate specificity. *Proceedings of the National Academy of Sciences of the United States of America*.

