# Supplementary material for: Synthesis and Antibacterial Activity of Quaternary Ammonium 4-Deoxypyridoxine Derivatives
Source: Biomed Res Int. 2016 Oct 5;2016:3864193. doi: 10.1155/2016/3864193 (PMC5069379; doi:10.1155/2016/3864193)
Supplement: Supplementary file 1 — The Supplementary Information contains synthetic procedures, analytical characteristics of compounds 4a-c and 5a-c, and biological experimental methods associated with this paper. [file 3864193.f1.doc]

**Supplementary Material**

**Synthesis and antibacterial activity of quaternary ammonium 4-deoxypyridoxine derivatives**

Nikita V. Shtyrlin^a^, Sergey V. Sapozhnikov^a^, Albina S. Galiullina^a^, Airat R. Kayumov^a^, Oksana V. Bondar^a^, Еlena P. Mirchink^b^, Еlena B. Isakova^b^, Аlexander А. Firsov^b^, Konstantin V. Balakin^a,c^ and Yurii G. Shtyrlina[[1]](#footnote-2)*

^a^ Kazan (Volga region) Federal University, Kremlyovskaya St. 18, Kazan, 420008, Russia

^b^ Gause Institute of New Antibiotics, Russian Academy of Medical Sciences, B. Pirogovskaya St. 11, Moscow, 119021, Russia

*^c^ Institute of physiologically active compounds of Russian Academy of Sciences, Severnyi pr. 1, Chernogolovka, Moscow reg., 142432, Russia*

The Electronic Supplementary Information contains synthetic procedures, analytical characteristics of compounds **4a-c** and **5a-c**, and biological experimental methods associated with this paper.

**Synthetic procedures**

**General information.** ^1^H and ^13^C NMR spectra were recorded on a Bruker AVANCE 400 spectrometer at operating frequency 400 and 101.56 MHz, respectively. Chemical shifts were measured with reference to the residual protons of the solvents (DMSO-d_6_, ^1^H, 2.50 ppm, ^13^C, 39.52 ppm; CDCl_3_, ^1^H, 7.26 ppm, ^13^C, 77.16 ppm; СD_3_OD, ^1^H, 4.87 ppm, ^13^C, 49.00 ppm). Coupling constants (J) are given in Hertz (Hz). The following abbreviations are used to describe coupling: s = singlet; d = doublet; t = triplet; m = multiplet; q=quartet, br s = broad singlet). Melting points were determined using a Stanford Research Systems MPA-100 OptiMelt melting point apparatus and are uncorrected. For TLC analysis silica gel plates from Sorbfil (Krasnodar, Russia) were used with UV light (254 nm/365 nm) or iron (III) chloride as developing agent. Column chromatography was performed on silica gel (60–200 mesh) from Acros.

HRMS mass spectra were obtained on a quadrupole time-of-flight (qTOF) AB Sciex Triple TOF 5600 mass spectrometer using turbo-ion spray source (nebulizer gas nitrogen, a positive ionization polarity, needle voltage 5500V). Recording of the spectra was performed in a TOF MS mode with a collision energy 10 eV, declustering potential 100 eV and with resolution more than 30 000 full-width half-maximum. Samples with the analytes concentration 5 μmol/l were prepared by dissolving of the test compounds in a mixture of methanol (HPLC-UV Grade, LabScan) and water (LC-MS Grade, Panreac) with a ratio of 1:1.

**General procedure for preparation of the quaternary ammonium salts 4a-c.** Tertiary amine (1 equiv.) was added to a solution of compound **3** (1 equiv.) in 30 mL of DMF. The reaction mixture was heated at 50^o^ C for 20 h, then the solvent was evaporated under reduced pressure. The product was recrystallized from acetone:diethyl ether mixture (10:1).

**N-((5-Acetoxy-4,6-dimethylpyridin-3-yl)methyl)-N,N-dimethyloctan-1-aminium chloride (4a).** The compound was obtained from **3** (150 mg, 0.70 mmol) and N,N-dimethyloctylamine (0.144 mL, 0.70 mmol) following the general procedure. Yield 54% (140 mg); white solid; mp 169–170 ^o^C (dec). ^1^H NMR (400 MHz, CDCl_3_, δ, ppm): 0.82 (t, ^3^J_нн_ = 6.8 Hz, CH_3_C_7_H_14_, 3H); 1.18–1.26 (m, 5СН_2_, 10Н); 1.72 (br s, СН_2_, 2Н); 2.34 (s, СН_3_, 3Н); 2.36 (s, СН_3_, 3Н); 2.43 (s, СН_3_, 3Н); 3.27 (s, 2CH_3_N^+^, 6H); 3.57–3.61 (m, CH_2_N^+^, 2H); 5.29 (s, CH_2_N^+^, 2H); 8.51 (s, СН_pyr_, 1Н). ^13^С NMR (100 MHz, CDCl_3_, δ, ppm): 14.08 (s, CH_3_); 14.43 (s, CH_3_); 19.68 (s, CH_3_); 20.50 (s, CH_2_); 22.59 (s, CH_2_); 23.00 (s, CH_2_); 26.39 (s, CH_2_); 29.05 (s, CH_2_); 29.26 (s, CH_2_); 31.64 (s, CH_2_); 49.26 (s, CH_3_N^+^); 62.97 (s, CH_2_N^+^); 63.72 (s, CH_2_N^+^); 122.04 (s, С_pyr_); 143.03 (s, С_pyr_); 145.55 (s, С_pyr_); 151.19 (s, С_pyr_); 153.83 (s, С_pyr_); 168.20 (s, С=O). HRМS-ESI: found [М–Cl]^+^ 335.2693, C_20_H_35_N_2_O_2_. Calculated [М– Cl]^+^ 335.2693.

**N-((5-Acetoxy-4,6-dimethylpyridin-3-yl)methyl)-N,N-dimethyldodecan-1-aminium chloride (4b).** The compound was obtained from **3** (175 mg, 0.82 mmol) and N,N-dimethyldodecylamine (0.222 mL, 0.82 mmol) following the general procedure. Yield 50% (175 mg); white solid; mp 169–170 ^o^C (dec). ^1^H NMR (400 MHz, CDCl_3_, δ, ppm): 0.84 (t, ^3^J_нн_ = 6.8 Hz, CH_3_C_11_H_22_, 3H); 1.20–1.28 (m, 9СН_2_, 18Н); 1.73 (br s, СН_2_, 2Н); 2.35 (s, СН_3_, 3Н); 2.37 (s, СН_3_, 3Н); 2.44 (s, СН_3_, 3Н); 3.28 (s, 2CH_3_N^+^, 6H); 3.57–3.61 (m, CH_2_N^+^, 2H); 5.29 (s, CH_2_N^+^, 2H); 8.51 (s, СН_pyr_, 1Н). ^13^С NMR (100 MHz, CDCl_3_, δ, ppm): 14.19 (s, CH_3_); 14.46 (s, CH_3_); 19.72 (s, CH_3_); 20.52 (s, CH_2_); 22.74 (s, CH_2_); 23.04 (s, CH_2_); 26.42 (s, CH_2_); 29.35 (s, CH_2_); 29.38 (s, CH_2_); 29.43 (s, CH_2_); 29.50 (s, CH_2_); 29.64 (s, CH_2_); 49.31 (s, CH_3_N^+^); 63.03 (s, CH_2_N^+^); 63.74 (s, CH_2_N^+^); 122.01 (s, С_pyr_); 143.06 (s, С_pyr_); 145.59 (s, С_pyr_); 151.20 (s, С_pyr_); 153.91 (s, С_pyr_); 168.23 (s, С=O). HRМS-ESI: found [М–Cl]^+^ 391.3319, C_25_H_43_N_2_O_2_. Calculated [М– Cl]^+^ 391.3319.

**N-((5-Acetoxy-4,6-dimethylpyridin-3-yl)methyl)-N,N-dimethyloctadecan-1-aminium chloride (4c).** The compound was obtained from **3** (150 mg, 0.70 mmol) and N,N-dimethyloctadecylamine (0.261 mL, 0.70 mmol) following the general procedure. Yield 53% (190 mg); white solid; mp 159–160 ^o^C (dec). ^1^H NMR (400 MHz, CDCl_3_, δ, ppm): 0.85 (t, ^3^J_нн_ = 6.7 Hz, CH_3_C_17_H_34_, 3H); 1.21–1.29 (m, 15СН_2_, 30Н); 1.74 (br s, СН_2_, 2Н); 2.36 (s, СН_3_, 3Н); 2.39 (s, СН_3_, 3Н); 2.46 (s, СН_3_, 3Н); 3.29 (s, 2CH_3_N^+^, 6H); 3.57–3.61 (m, CH_2_N^+^, 2H); 5.30 (s, CH_2_N^+^, 2H); 8.50 (s, СН_pyr_, 1Н). ^13^С NMR (100 MHz, CDCl_3_, δ, ppm): 14.22 (s, CH_3_); 14.51 (s, CH_3_); 19.75 (s, CH_3_); 20.55 (s, CH_2_); 22.79 (s, CH_2_); 23.07 (s, CH_2_); 26.44 (s, CH_2_); 29.39 (s, CH_2_); 29.46 (s, CH_2_); 29.55 (s, CH_2_); 29.69 (s, CH_2_); 29.76 (s, CH_2_); 29.80 (s, CH_2_); 32.02 (s, CH_2_); 49.36 (s, CH_3_N^+^); 63.10 (s, CH_2_N^+^); 63.79 (s, CH_2_N^+^); 122.01 (s, С_pyr_); 143.13 (s, С_pyr_); 145.64 (s, С_pyr_); 153.18 (s, С_pyr_); 153.98 (s, С_pyr_); 168.26 (s, С=O). HRМS-ESI: found [М–Cl]^+^ 475.4258, C_30_H_55_N_2_O_2_. Calculated [М– Cl]^+^ 475.4258.

**General procedure for preparation of 5a-c.** A mixture of quaternary ammonium salt **4a-c** (1 equiv) and 1 mL of concentrated HCl in 20 mL of water was stirred at 60 ^o^C for 24 h. The solvent was evaporated under reduced pressure to obtain **5a-c** in quantitative yield.

**5-((Octyldimethylammonio)methyl)-3-hydroxy-2,4-dimethylpyridin-1-ium dichloride (5a).** The compound was obtained from **4a** (100 mg, 0.27 mmol) following the general procedure. Yield quantitative (0.99 mg); white solid; mp 186–188 ^o^C (dec.). ^1^H NMR (400 MHz, DMSO-d_6_, δ, ppm): 0.87 (t, ^3^J_нн_ = 6.8 Hz, CH_3_C_11_H_22_, 3H); 1.27–1.31 (m, 5СН_2_, 5Н);1.77 (br s, СН_2_, 2Н); 2.50 (s, СН_3_, 3Н); 2.68 (s, СН_3_, 3Н); 2.99 (s, 2CH_3_N^+^, 6H); 3.42–3.46 (m, CH_2_N^+^, 2H); 4.78 (s, CH_2_N^+^, 2H); 8.49 (s, СН_pyr_, 1Н); 10.96 (s, OH, 1H). ^13^С NMR (100 MHz, DMSO-d_6_, δ, ppm): 13.94 (s, CH_3_); 14.87 (s, CH_3_); 15.43 (s, CH_3_); 21.83 (s, CH_2_); 22.03 (s, CH_2_); 25.84 (s, CH_2_); 28.41 (s, CH_2_); 28.48 (s, CH_2_); 31.14 (s, CH_2_); 48.66 (s, CH_3_N^+^); 60.43 (s, CH_2_N^+^); 64.48 (s, CH_2_N^+^); 124.81 (s, С_pyr_); 136.27 (s, С_pyr_); 142.15 (s, С_pyr_); 152.87 (s, С_pyr_). HRМS-ESI: found [М–Cl]^+^ 293.2587, C_18_H_34_N_2_O. Calculated [М– Cl]^+^ 293.2587.

**5-((Dodecyldimethylammonio)methyl)-3-hydroxy-2,4-dimethylpyridin-1-ium dichloride (5b).** The compound was obtained from **4b** (100 mg, 0.23 mmol) following the general procedure. Yield quantitative (0.99 mg); white solid; mp 187–188 ^o^C (dec.). ^1^H NMR (400 MHz, CD_3_OD, δ, ppm): 0.88 (t, ^3^J_нн_ = 6.7 Hz, CH_3_C_11_H_22_, 3H); 1.28–1.42 (m, 18СН_2_, 9Н);1.90 (br s, СН_2_, 2Н); 2.60 (s, СН_3_, 3Н); 2.70 (s, СН_3_, 3Н); 3.09 (s, 2CH_3_N^+^, 6H); 3.50 (br s, CH_2_N^+^, 2H); 4.82 (s, CH_2_N^+^, 2H); 8.49 (s, СН_pyr_, 1Н). ^13^С NMR (100 MHz, CD_3_OD, δ, ppm): 14.43 (s, CH_3_); 15.47 (s, CH_3_); 15.63 (s, CH_3_); 23.73 (s, CH_2_); 23.81 (s, CH_2_); 27.50 (s, CH_2_); 30.32 (s, CH_2_); 30.46 (s, CH_2_); 30.58 (s, CH_2_); 30.65 (s, CH_2_); 30.74 (s, CH_2_); 33.06 (s, CH_2_); 50.29 (s, CH_3_N^+^); 62.69 (s, CH_2_N^+^); 67.40 (s, CH_2_N^+^); 126.59 (s, С_pyr_); 136.93 (s, С_pyr_); 143.83 (s, С_pyr_); 148.57 (s, С_pyr_); 155.46 (s, С_pyr_). HRМS-ESI: found [М–H–2Cl]^+^ 349.3213, C_22_H_42_N_2_O. Calculated [М–H–2Cl]^+^ 349.3213.

**5-((Dimethyl(octadecyl)ammonio)methyl)-3-hydroxy-2,4-dimethylpyridin-1-ium dichloride (5c).** The compound was obtained from **4c** (100 mg, 0.20 mmol) following the general procedure. Yield quantitative (0.99 mg); white solid; mp 185–187 ^o^C (dec.). ^1^H NMR (400 MHz, DMSO-d_6_, δ, ppm): 0.85 (t, ^3^J_нн_ = 6.7 Hz, CH_3_C_17_H_34_, 3H); 1.23–1.33 (m, 15СН_2_, 30Н);1.77 (br s, СН_2_, 2Н); 2.50 (s, СН_3_, 3Н); 2.68 (s, СН_3_, 3Н); 2.99 (s, 2CH_3_N^+^, 6H); 3.42–3.46 (m, CH_2_N^+^, 2H); 4.77 (s, CH_2_N^+^, 2H); 8.49 (s, СН_pyr_, 1Н); 10.94 (s, OH, 1H). ^13^С NMR (100 MHz, DMSO-d_6_, δ, ppm): 13.94 (s, CH_3_); 14.86 (s, CH_3_); 15.46 (s, CH_3_); 21.85 (s, CH_2_); 22.08 (s, CH_2_); 25.85 (s, CH_2_); 28.56 (s, CH_2_); 28.68 (s, CH_2_); 28.79 (s, CH_2_); 29.03 (s, CH_2_); 31.27 (s, CH_2_); 48.66 (s, CH_3_N^+^); 60.42 (s, CH_2_N^+^); 64.50 (s, CH_2_N^+^); 124.81 (s, С_pyr_); 136.29 (s, С_pyr_); 142.17 (s, С_pyr_); 152.87 (s, С_pyr_). HRМS-ESI: found [М–H–2Cl]^+^ 433.4152, C_28_H_54_N_2_O. Calculated [М–H–2Cl]^+^ 433.4152.

**Biological experiments**

**Antibacterial activity.** The antibacterial activity of compounds was evaluated on the number of gram-positive (*Staphylococcus aureus* ATCC® 29213™, *Staphylococcus epidermidis* (clinical isolate), *Micrococcus luteus* (clinical isolate), *Bacillus subtilis* 168) and gram-negative bacteria (*Escherichia coli* АТСС® 25922™, *Pseudomonas aeruginosa* АТСС® 27853™, *Salmonella typhymurium TA100*). Clinical isolates of *Staphylococcus* *epidermidis* and *Micrococcus luteus* were obtained from the Kazan Institute of Epidemiology and Microbiology (Kazan, Russia). The antibacterial activity of compounds **4c** and **5c** was additionally evaluated on *Staphylococcus aureus* ATCC® 700699™ and a number of clinical isolates: *Staphylococcus aureus* 100 MRSA, *Staphylococcus aureus* 5 MRSA, *Staphylococcus aureus* 6 MRSA, *Staphylococcus aureus* 3797 MRSA, *Staphylococcus aureus* 3798 MRSA, *Staphylococcus aureus* 4603 MRSA, *Staphylococcus haemoliticus* 161, *Staphylococcus haemoliticus* 1025, *Staphylococcus haemoliticus* 602, *Staphylococcus haemoliticus* 585, *Staphylococcus epidermidis* 681, *Staphylococcus epidermidis* 9, *Enterococcus faecalis* 560, *Enterococcus faecium* 569, *Escherichia coli* 396, *Pseudomonas aeruginosa* 43.

The MICs of compounds were determined by the broth microdilution method in Mueller-Hinton (MH) broth (pH=7.3) in 96-well plates. The compounds were diluted to a final concentrations ranging from 0.5 to 1000 µg/mL for the initial screening and 64–0.5 μg/mL while testing of **4c** and **5c** on clinical isolate strains. The bacterial suspension (2-9×10^4^ CFU/mL) 200-µl aliquots were seeded into 96-well plates and their incubation was followed. The MIC was determined as the lowest concentration of compound for which no visible bacterial growth could be observed after 24 h of incubation at 37 °C.

**Cytotoxic activity.** Human skin fibroblasts (HSFs) were isolated from the skin explant according to the conventional protocol [1s]. HEK 293 (human embryonic kidney) cells were obtained from the ATCC collection. HSFs cells were cultured in the minimum essential medium Eagle (α-MEM) supplemented with 10% fetal bovine serum, 2 mM Lglutamine, 100 µg/mL streptomycin and 100 U/mL penicillin under standard conditions (37 °C, 5% CO_2_ atmosphere). HEK 293 cells were grown in the same conditions, but in the Dulbecco’s modified Eagle’s medium (DMEM). Adhered cells were collected from the culture flask bydetaching them with trypsin–EDTA solution. Suspended cells were washed by centrifugation at 200g in PBS.

Cytotoxic concentrations (IC_50_) of compounds were determined with the use of MTT assay. Cells were pre-seeded in 96-well plate at the density of 1000-2000 cells per well and cultured with adding a series of diluted water solutions of compounds for 3 days under standard conditions. Culture medium in the plate was then replaced by the fresh one supplemented with 0.5 mg/mL MTT and additionally kept for 4 h to allow for reduction of MTT into colored product (formazan) by metabolically active cells. Optical absorbance of produced formazan, proportional to viable cell number, was registered on Infinite 200 PRO analyzer at 550 nm.

**Genotoxicity.** *S. typhimurium* strain TA100 [2s] was grown overnight in 5 ml of LB medium, diluted 4-times by pre-warmed LB and incubation was continued for 2 h. Cells were harvested, washed once by 1× salt base solution (g/L: Sodium citrate×3Н_2_О – 0.5; К_2_НРО_3_×3Н_2_О – 14; КН_2_РО_3_ – 6; (NH_4_)_2_SO_4_ – 1; MgSO_4_×7Н_2_О – 0.5) and resuspended in 6 mL of 1× salt base. 100 μL of bacterial suspension was mixed with top agar (0.5% agar, 0.5% NaCl, 50 mM L-histidine, 50 mM biotin, pH 7.4, 42 ºC) in a final volume of 3 mL and the substance to be tested. Each mixture was then seeded onto the minimal agar plates (1.5% agar in the 1X salt base supplemented with 0.5% glucose and ampicillin 10 µg/mL). Next the plates were incubated at 37 ºC for 72 hours and colonies were counted. Sodium azide (10 µg/mL) was used as a positive control.

The SOS chromotest was performed by using the *Salmonella typhimurium TA1535/pSK1002* as described in [3s]. Briefly, aliquots of 0.5 ml of an overnight culture of the tester strains were diluted in 5 ml of LB medium and then incubated with rigorous agitation in presence of the ficin substances. The Mytomycin C (Sigma) at concentration of 1 µg/mL was used as a positive control. After 4 h of incubation, the cell density (A600) and the β-galactosidase activity was measured by the Miller’s protocol [4s] with modifications. Cells were harvested from 0.5-1.5 mL of culture liquid, resuspended in 800 µL of Z-buffer [60 mM Na_2_HPO_4_ • 7H_2_O, 40 mM NaH_2_PO_4_ • H_2_O, 10 mM KCl, and 1 mM MgSO_4_ • 7H_2_O (pH 7.0)] containing additionally 0.005% cetyl trimethylammonium bromide (CTAB) and 50 mM β-mercaptoethanol was added. After preincubation at 30 ^o^C for 5 min, the reaction was started by adding of 200 µL of 4 mg/mL o-nitrophenyl-β-D-galactopyranoside in Z-buffer. When the yellow color appeared, the reaction was stopped by 500 µL of 1M Na_2_CO_3_. For the blank solution, the Na_2_CO_3_ was added prior the incubation. The β-galactosidase activity was measured at A420 nm. To calculate the Miller units, we used the following formula: [A420/(A600 of 1:10 dilution of cells × time of incubation)] × 1000.

**References**

[1s] L. Rittié, and G. J. Fisher, “Isolation and culture of skin fibroblasts”, *Methods in Molecular Medicine,* vol. 117, pp. 83–98,2005.

[2s] Y. Oda, S. Nakamura, I. Oki, T. Kato and H. Shinagawa, “Evaluation of the new system (*umu*-test) for the detection of environmental mutagens and carcinogens”, *Mutation Research*, vol. 147, no. 5, pp. 219–229, 1985.

[3s] J. McCann and B. N. Ames, “A simple method for detecting environmental carcinogens as mutagens”, *Annals of the New York Academy of Sciences*, vol. 271, pp. 5–13, 1976.

[4s] J. H. Miller, Experiments in Molecular Genetics, N.Y.: Cold Spring Harbor Lab., 1972, pp. 352–355.

1.  Corresponding author. Tel.: +7-843-233-7363; fax: +7-843-233-7531; e-mail: yurii.shtyrlin@gmail.com [↑](#footnote-ref-2)
